# SARS-CoV-2 in Namibian Dogs

**DOI:** 10.3390/vaccines10122134

**Published:** 2022-12-13

**Authors:** Umberto Molini, Lauren M. Coetzee, Tanya Engelbrecht, Lourens de Villiers, Mari de Villiers, Iolanda Mangone, Valentina Curini, Siegfried Khaiseb, Massimo Ancora, Cesare Cammà, Alessio Lorusso, Giovanni Franzo

**Affiliations:** 1 School of Veterinary Medicine, Faculty of Health Sciences and Veterinary Medicine, University of Namibia, Neudamm Campus, Private Bag 13301, Windhoek 9000, Namibia; 2 Central Veterinary Laboratory, 24 Goethe Street, Private Bag 13301, Windhoek 9000, Namibia; 3 Istituto Zooprofilattico Sperimentale dell’Abruzzo e del Molise, 64100 Teramo, Italy; 4 Dept. of Animal Medicine, Production and Health, University of Padova, Viale dell’Università 16, 35020 Legnaro, Italy

**Keywords:** SARS-CoV-2, dog, Namibia, diagnosis, sequencing

## Abstract

The pandemic of coronavirus disease 19 (COVID-19) has focused the attention of researchers, and especially public opinion, on the role of the human-animal-environment interface in disease emergence. At the beginning of the COVID-19 pandemic, media reports regarding the role of pets in severe acute respiratory syndrome coronavirus 2 (SARS-CoV-2) caused significant concern and social anxiety. Although nowadays proven negligible in developed countries, essentially no studies have been performed in low-income African areas where companion animals are often raised differently from high income countries, and the contact patterns occurring in these scenarios could affect the epidemiological scenario. An extensive molecular biology survey was performed from March 2022 to September 2022 on Namibian dogs residing in urban and rural areas, showing a low but not negligible SARS-CoV-2 prevalence (1%; 95CI: 0.33–2.32%) of 5 out of 500. In only one instance (i.e., a 4-year-old female Labrador) was there a clear association that could be established between the infections of the owner and animal. In all other cases, no evidence of human infection could be obtained and no episodes of COVID-19 were reported by the owners. Although no consistent evidence of pet-to-pet transmission was proven in the present study, a cautionary principle suggests intensive and dedicated investigation into companion animal populations, especially when animal contact is frequent and a particularly susceptible population is present.

## 1. Introduction

The pandemic of coronavirus disease 19 (COVID-19) caused by severe acute respiratory syndrome coronavirus 2 (SARS-CoV-2) has focused the attention of researchers, and especially public opinion, on the role of the human-animal-environment interface in new disease emergence [1,2,3]. Although bats and wild animals were the most likely source of spillover [4], other species, including domestic ones, were proven to be susceptible [5,6]. Companion animals were clearly of particular concern because of their high numbers and close contact with human beings. Currently, SARS-CoV-2 has been demonstrated to infect pets in several American, Asian and European countries [5,7]. However, essentially no study has been performed on African companion animals, especially in sub-Saharan regions [8]. Current evidence suggests that transmission is essentially unidirectional, via human-to-pet transmission events, although animal-to-animal and animal-to-human transmission events were suggested in certain outbreaks [5]. Remarkably, companion animals in developing countries are often raised differently from high income countries, and the contact patterns occurring in these scenarios could affect the epidemiological scenario. Having this in mind, an extensive survey was performed on Namibian dogs residing in urban and rural areas.

## 2. Materials and Methods

From March 2022 to September 2022, blood samples (499) and oropharyngeal and nasal swabs (10) were collected from 500 dogs around 8 regions of Namibia (Table 1).

The subjects included in this study belonged to two categories, depending on the knowledge of the owner’s health status. Ten dogs were sampled from Windhoek, Khomas Region, from owners who tested positive for SARS-CoV-2. The remaining ones were included in this study to assess SARS-CoV-2 circulation in the Namibian dog population, especially outdoor living pets from low-income areas. For this purpose, animals were randomly selected during the vaccination and prevention campaign performed around Namibia by the mobile clinic of the University of Namibia. All the samples after collection were refrigerated and sent to the Central Veterinary Laboratory in Windhoek. Total genomic RNA was carried out using a High Pure Viral Nucleic Acid Kit (Hoffman, Switzerland) with an elution volume of 100 μL following the manufacturer’s instructions. Purified RNA was tested for SARS-CoV-2 by means of the LightMix^®^ SarbecoV E-gene plus EAV (TIB MOLBIOL, Berlin, Germany). An aliquot of positive samples with a cut-off for positivity of CT ≤ 25 was sent to the Istituto Zooprofilattico Sperimentale dell’Abruzzo e Molise (IZS), Italy, for whole genome sequencing (WGS) using the COVIDSeq Test (Illumina Inc., San Diego, CA, USA). This library preparation method was automated in-house and validated with the Hamilton Microlab STAR Liquid Handling System (Hamilton Robotics, Reno, NV, USA) at the “National Reference Centre for Whole-Genome Sequencing of microbial pathogens: database and bioinformatic analysis” (GENPAT) at IZS in Teramo. Deep sequencing was performed with the NextSeq 500 platform (Illumina Inc., San Diego, CA, USA) using the NextSeq 500/550 Mid Output Reagent Cartridge v2 with 300 cycles and standard 150 bp paired-end reads. After quality control and trimming of the reads using FastQC and Trimmomatic [9], mapping to the Wuhan-Hu-1 reference genome (accension number NC_045512) was performed with the BWA tool [10], and the consensus sequence was obtained using iVar (v1.3.1) (intrahost variant analysis of replicates; github.com/andersenlab/ ivar; accessed on 13 August 2022). All analysis steps were automatically performed at the end of the sequencing run using the GENPAT platform at IZS in Teramo as described in [11]. The identification of the occurring SARS-CoV-2 lineage was confirmed by using the PANGO tool via the web (https://pangolin.cog-uk.io/; (accessed on 10 November 2022)).

## 3. Results

Out of the 500 dogs tested, 5 were positive for COVID-19 (Table 1).

Only 1 out of the 10 dogs (10%; 95CI: 0.025–44.5%) whose owners tested SARS-CoV-2 positive was also infected: a 4-year-old female Labrador without any clinical signs (Ct 19.22) from Windhoek, Khomas Region.

Out of the 490 animals included in the larger survey, 4 (0.8%; 95CI: 0.02–2.08%) were SARS-CoV-2 positive: a 2-year-old female Jack Russell (Ct 34.61) from Windhoek; a 1-year-old, mixed-breed male from Outjo, Kunene Region, that presented loss of appetite, oculonasal discharge, lymphadenomegaly (submandibular and prescapular lymph nodes) and splenomegaly (Ct 33.16); and 2 dogs from Gobabis, Omaheke Region, one of which was a 1-year-old, mixed-breed female that presented loss of appetite, weight loss and dry and pale mucous membranes (Ct 36.88), and the other a 7-months-old, mixed-breed male that also presented lymphadenomegaly (prescapular and popliteal lymph nodes) (Ct 37). WGS was performed only on the Labrador that presented a Ct of 19.22. The sequencing run produced a total number of 3164700 raw reads with an average quality score of 33 per sample. Out of them, 619237 reads were mapped to the Wuhan-Hu-1 reference genome with an average depth coverage of 3042. The consensus sequence obtained belonged to the BA.2 PANGO lineage (last accessed on 10 November 2022), a sub-lineage of the Omicron VOC. A complete report of the variant features and distribution is available at https://cov-lineages.org/lineage.html?lineage=BA.2 (accessed on 22 November 2022). The sequence has been released to GenBank under accession number OP809597.

## 4. Discussion

The present study’s results demonstrate a low SARS-CoV-2 infection rate (1%; 95CI: 0.33–2.32%) in Namibian dogs, largely in agreement with the previous reports obtained from high-income countries [12]. In only one instance (i.e., a 4-year-old female Labrador) was there a clear association that could be established between the infections of the owner and animal, since the animal belonged to a woman who, requiring medical assistance, was tested in presence of overt clinical signs. The animal was only sampled a posteriori, based on human test results. It is noteworthy that this was the only subject that featured a high viral titre, which may suggest the relevance of close contact for the occurrence of an effective infection establishment in dogs. Accordingly, the infection frequency was remarkably higher in the dogs living with infected owners compared to the general population, for which no information on the owner’s status was available. Although no respective human sequence could be obtained, since sequencing is not routinely performed under the Namibia diagnostic protocol, the detected strain was the most prevalent in Namibia and neighbouring countries at that time, further supporting the current evidence on human-to-pet transmission (see https://cov-lineages.org/lineage.html?lineage=BA.2 accessed on 22 November 2022).

In the other sampled pet population, no evidence of human infection could be obtained and no episodes of COVID-19 were reported by the owners. However, we must stress that this was not the aim of our study and its design was thus not adequate for this purpose. Moreover, the samples were obtained from rural areas with extremely limited access to healthcare services and where the people’s awareness of COVID-19 was scant. Finally, human subclinical infection is common and cannot be excluded from the involved animal owners. Rare evidence of relevant pet-to-human or pet-to-pet transmission has been reported in other countries where companion animal infections have been extensively investigated, and dogs did not seem to be involved [5]. Pets in high-income countries often have limited contact opportunities, which could determine the negligible SARS-CoV-2 prevalence in the absence of the owner’s infection. On the contrary, the investigated population has an outdoor life and thus a denser contact network compared to indoor pets. Therefore, a certain among-animals transmission cannot be excluded and might contribute to viral spread, potentially to human beings also. No evidence of such a phenomenon could be obtained in the present study due to its design. However, even if the implications on public health are likely reduced, a cautionary principle suggests that further studies should be performed to investigate the occurrence of such transmission patterns in light of the peculiar epidemiological scenario of degraded urban areas and considering the potential consequences for a fragile and immunocompromised human population, being that HIV prevalence in Namibia is among the highest in the world [13,14,15].

Interestingly, three out of five dogs showed overt clinical signs, as reported by other authors [7]. However, a causal association with SARS-CoV-2 could not be confidentially stated, and the high Ct observed in those animals suggests an incidental finding rather than an actual etiological role. If other infections or poor animal conditions were responsible for clinical signs [16] or favored SARS-CoV-2 infection, then this would require further investigation.

## 5. Conclusions

Overall, a low but not negligible prevalence in Namibian free-range outdoor dogs was reported, often in the absence of a clear association with the owner’s infection. No consistent evidence of pet-to-pet transmission could be proven in the present study. Nevertheless, a cautionary principle suggests intensive and dedicated investigation into companion animal populations, especially when animal contacts are frequent and a particularly susceptible population is present.

## Figures and Tables

**Table 1:** Summary of samples’ origins, features and results.

Region	N. dogs	Blood Samples	Oral-Nasal Swabs	SARS-CoV-2 Positive
Erongo	43	43	0	0
Hardap	68	68	0	0
Karas	44	44	0	0
Kavango	58	58	0	0
Kunene	73	73	0	1
Khomas	59	58	10	2
Omaheke	83	83	0	2
Otjozondjupa	72	72	0	0

## Data Availability

Not applicable.
